# Macrophages and cardiac lesion in zebrafish: what can single-cell RNA sequencing reveal?

**DOI:** 10.3389/fcvm.2025.1570582

**Published:** 2025-04-11

**Authors:** Rebeca Bosso dos Santos Luz, André Guilherme Portela Paula, Andressa Pacheco Czaikovski, Bruno Sime Ferreira Nunes, Jordana Dinora De Lima, Lais Cavalieri Paredes, Thais Sibioni Berti Bastos, Rebecca Richardson, Tarcio Teodoro Braga

**Affiliations:** ^1^ Basic Pathology Department, Biological Sciences Sector, Federal University of Paraná, Curitiba, Brazil; ^2^ Biological Sciences Institute IV, University of São Paulo, São Paulo, Brazil; ^3^ School of Physiology, Pharmacology & Neuroscience, Faculty of Biomedical Sciences, University of Bristol, Bristol, United Kingdom

**Keywords:** zebrafish, macrophages, cryoinjury, cardiac resection, regeneration, fibrosis, repair, single-cell RNA sequencing

## Abstract

Unlike mammals, zebrafish can regenerate their heart after cardiac insult. There are several ways to perform cardiac injury in zebrafish, but cryoinjury most closely resembles human myocardial infarction (MI). Studies demonstrated that macrophages are essential cells from the beginning to later stages of cardiac injury throughout the regenerative process in zebrafish. These cells have phenotypic plasticity; hence, overly sensitive techniques, such as single-cell RNA sequencing (scRNAseq), are essential for uncovering the phenotype needed for zebrafish cardiac injury regeneration, from inflammatory profile initiation to scar resolution. This technique enables the RNA sequencing of individual cells, thus generating clusters of cells with similar gene expression and allowing the study of a particular cell population. Therefore, in this review, we focused on discussing data obtained by scRNAseq of macrophages in the context of cardiac injury. We found that from 1 to 7 days post-injury (dpi), macrophages are present with inflammatory and reparative functions in either cryoinjury or ventricular resection. At 14 dpi, there were differences between the injury models, especially in the expression profile of inflammatory cytokines, and studies with later time points are needed to understand the gene expression that enrolls the collagen scar resorption dynamic.

## Introduction

**Figure 1:**
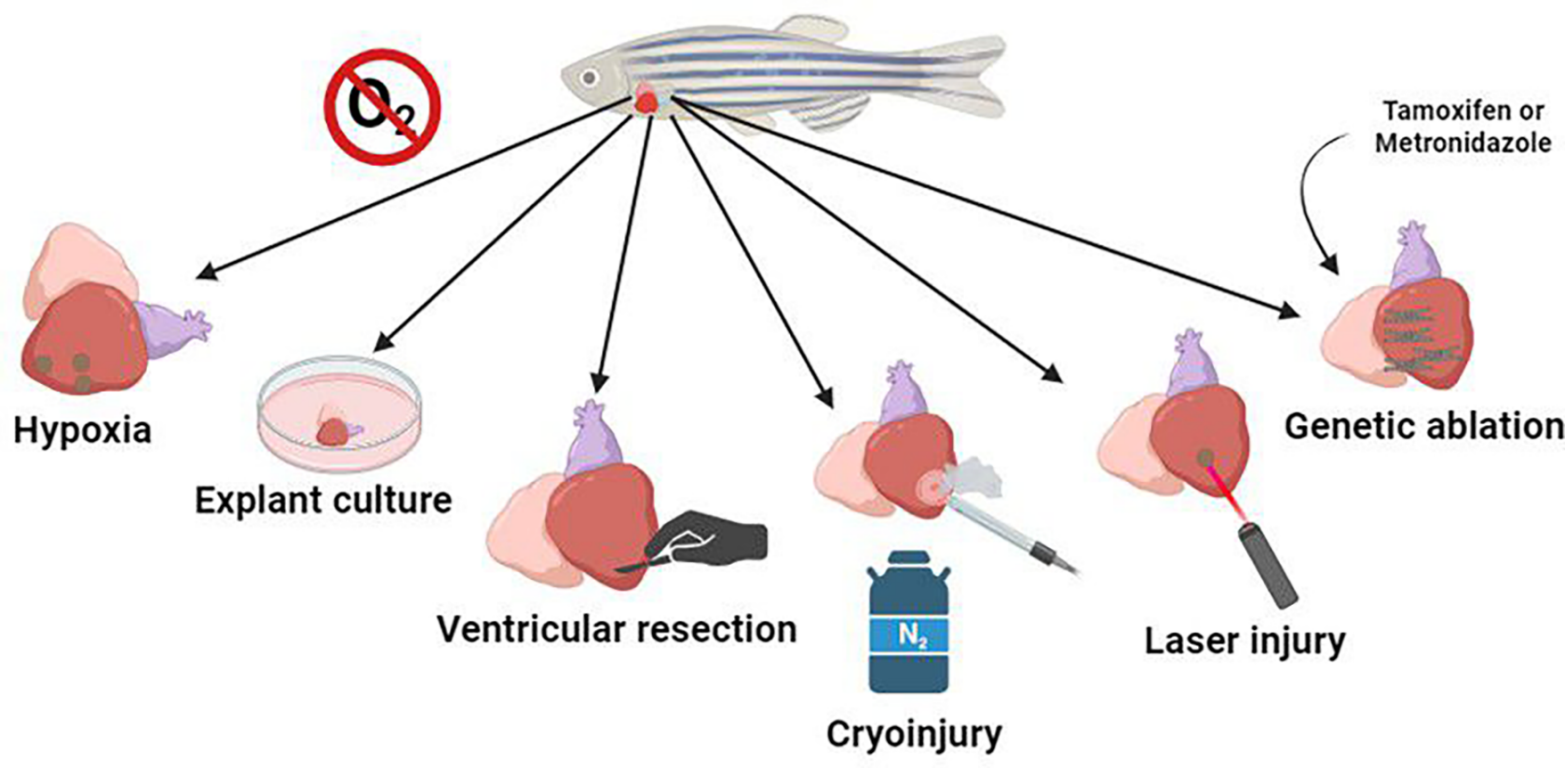
The most commonly used means of mimicking human heart disease are hypoxia, where the animal's habitat is deprived of oxygen, leading to lesions; explant culture, where the animal's organ is kept in culture, and it is possible to observe cell-extracellular matrix interactions in regeneration; ventricular resection, to mark the mechanisms and expression of genes in the regeneration process; cryoinjury, which uses a cryo-cooled probe pressed on the myocardium to mimic an infarction; laser injury, which generates selective damage in the region of interest; and genetic ablation, in which cardiomyocytes are genetically modified to generate cytotoxic metabolites when activated via a prodrug, causing mosaic cell death in a temporally controlled manner.

Heart infarction is characterized by the interruption of oxygenated blood supply to part of the cardiac tissue, with this clinical condition being the main cause of heart failure, a syndrome that has high prevalence, incidence, and mortality (1). It also negatively affects life quality and presents high health costs (1). These characteristics are highlighted by the fact that after injury, the human heart has little capacity for regeneration. Upon injury, there is a substantial formation of fibrin scars that alter the functionality of the tissue (2). Therefore, the search for mechanisms that resolve heart injury or even for mechanisms that generate total healing of the myocardium remains of high clinical importance. Unlike humans, adult zebrafish (*Danio rerio*) can regenerate the heart within two to three months of a ventricular resection of up to 20% of the myocardium (3). Zebrafish cardiomyocytes, especially those in the epicardial borders, can dedifferentiate and proliferate, resulting in the remodeling of the new tissue (3). Recent methodologies to study the mechanisms of zebrafish myocardial regeneration have been used to better understand human infarction (4) (Figure 1, Table 1).

**Table 1:** Comparison of cardiac injury models in zebrafish.

Characteristic	Ventricular resection	Cryoinjury	Genetic ablation	Hypoxia
Injury (%)	20	25–30	60	–
Cell death	Apoptosis	Apoptosis	Apoptosis	Apoptosis
Cardiac tissue specificity	Yes	Yes	Yes	No
Localized injury	Yes	Yes	No	No
Fibrosis	Reduced	High	High	Reduced
Ventricular remodeling	Reduced	High	Reduced	Reduced
Functional recovery	High	High	High	High
Regeneration time	30–60 days	80–130 days	30 days	–
Transgenic animals	No	No	Yes	No
Use of larvae	No	No	Yes	Yes

Among all cells involved in heart regeneration in zebrafish and in cardiac fibrosis in mammals (5) macrophages coordinate all the cardiac repair phases (6). In contrast, the adult mammalian heart has limited regenerative capacity. Nevertheless, the healing of infarcted myocardium similarly depends on a well-orchestrated sequence of cellular events, including inflammatory, proliferative, and maturation phases. Mononuclear cells and macrophages are particularly crucial during all repair phases, including fibrosis, highlighting their essential role in myocardial repair (5). Thus, they could be eligible targets for reversion, treatment, or prevention of human cardiac fibrosis resulting from myocardial infarction (MI). However, these cells have high plasticity and different phenotypes during cardiac repair, and it is therefore necessary to understand the mechanisms behind these phenotypic changes. One approach used to identify cellular markers and potential drug targets is through transcriptomic analysis. This methodology enables the characterization of cell phenotypes by analyzing a pool of cells (bulk RNA sequencing) or in a single-cell manner (scRNA sequencing). Several studies have attempted to differentiate macrophage subpopulations using different methodologies and experimental designs (6–10) and therefore, in this review, we compile these studies and focus on cardiac injury recovery, paying special attention to macrophages as crucial cells that regulate the fate of the injured heart tissue.

## Cardiac lesion models in zebrafish

The zebrafish is a teleost fish that can regenerates all organs and appendices. Therefore, it can be used to uncover the mechanisms by which it regenerates its heart, providing researchers the knowledge to propose new therapeutic targets able to undo or prevent human cardiac fibrosis. There are several ways to study MI repair and regeneration using zebrafish as a model, these models are outlined below.

### Hypoxia

Hypoxia is a consequence of MI, it generates reactive oxygen species (ROS), leading to inflammation in zebrafish and in mammals. Although zebrafish are used to low oxygen environments (11), hypoxia can induce cardiac inflammatory responses. After 14 h of exposure to a hypoxic environment, there is cardiomyocyte apoptosis, while necrosis is observed after 18 h, together with the recruitment of neutrophils and macrophages (12). This is a model that mimics what happens in MI, but it does not result in scar deposition.

### Ventricular resection

Ventricular resection is the surgical removal of 20% of the zebrafish ventricle (3), it was the method by which Kenneth Poss and colleagues, in 2002, described zebrafish heart regeneration, it can regenerate the entire lost region within 60 days after injury. After ventricular resection, thrombosis occurs, and a fibrin cloth is formed within four days of resection. From 7 to 14 days of injury, cardiomyocytes repopulate the sectioned area (13), a collagen fibrotic scar covers the injured tissue and at 21 dpi, the zebrafish starts to regenerate the scar, it is replaced by functional cardiomyocytes and the collagen is reabsorbed (3).

### Cryoinjury

The cryoinjury method was established by Chablais et al. in 2011 (14). This method uses a supercooled metal probe pressed to the zebrafish ventricle, which generates pathological changes like those caused by MI in mammals. As a result, an inflammatory response activates epicardial and endocardial cells and fibrotic tissue is deposited, which replaces the necrotic tissue (15). The resolution is activated following this process, where cardiomyocyte proliferation occurs, repopulating the injury site and leading to cardiac homeostasis, regeneration, and restoration (15). The emerging scar is wholly resolved after 130 days of injury (16).

After the cardiac injury, zebrafish express the *vegfc* gene, which promotes the proliferation of cardiac endothelial cells (cECs). *Vegfc* signaling activates the expression of *emilin2a*, a pro-regenerative factor for cECs and scar resolution. The *emilin2a* gene induces the expression of the chemokine *cxcl8a* in epicardial cells while regenerating cECs express *cxcr1* receptors. Therefore, the interaction of *cxcl8a-cxcr1* signaling aids in revascularizing injured tissue (17).

### Laser injury

Unlike techniques that use drugs or even genetics to mimic cardiac lesions, the lesion caused by the laser is more specific, reducing tissue damage in unlikely areas. Laser injury is a technique used in the zebrafish model, and organ regeneration and functionality have been observed in larvae (18, 19). Reduced cardiac performance was observed in larvae two hours after heart lesion by laser, as well as bradycardia and minor bleeding and apoptosis and necrosis were observed in the lesion. After 24 h, the heart resolved the injury, with re-established functional performance (20).

### Explant culture

This technique is used in *ex vivo* cultures to study the gene expression of epicardial and endocardial cells in infarction models. It is, therefore, a methodology to study the signaling involved in the dynamics of development and recovery of the heart post-injury, and not a *per se* cardiac injury methodology. The method's strength is that it characterizes epicardial cell interactions with the extracellular matrix in regenerative processes (21). Epicardial cells need 48 h of culture before experimental use, and the culture can be maintained for up to 6 days (22). This protocol is done by extracting the heart of the newly injured zebrafish and explanting it in fibrin gels in a tissue culture plate to mimic the formation of blood clots, which is essential in the migration of cells upon injury.

### Genetic modifications

Cardiac cells can be genetically modified to present phenotypes related to cardiac hyperplasia, hypertrophy and/or dysfunction (23). The exacerbated ablation of cardiomyocytes modifies the electrical conduction of the organ, mimicking the symptoms of heart failure (24). Mutations can be made by silencing genes, morpholino knockdown with or without TALEN genome editing, knockout and CRISPR-Cas9 technology. These modifications can delete specific genes that affects the cardiac tissue structure or alters the cardiac function, targeting signal transduction pathways, for example. Some targeted genes are highlighted at the Table 2, more information about it can be found at Narumanchi et al., 2021 review (23).

**Table 2:** Central genes studied in zebrafish heart regeneration.

Main genes used in the genetic ablation of the zebrafish heart		
Gene	Function	Reference
*cmlc1*	A gene involved in cardiac contraction, cardiac morphogenesis, and myofibril assembly.	National Library of Medicine (https://www.ncbi.nlm.nih.gov/gene/64671)
*cmlc2*	A gene involved in cardiac muscle cell proliferation, heart contraction, and myofibril assembly.	National Library of Medicine (https://www.ncbi.nlm.nih.gov/gene/30592)
*BAG3*	Expressed in slow and fast muscles and the heart, it is used to study heart disease and muscular dystrophy.	National Library of Medicine (https://www.ncbi.nlm.nih.gov/gene/445139)
*tbx5a*	Involved in cardiac development and regeneration of heart muscle tissue.	National Library of Medicine (https://pubmed.ncbi.nlm.nih.gov/29382818)
*erbb4a*	They are expressed in the nervous system and heart, acting in the formation of neurons.	National Library of Medicine (https://www.ncbi.nlm.nih.gov/gene/100007677)
*nfatc1*	Involved in the morphogenesis of the atrioventricular valve.	National Library of Medicine (https://www.ncbi.nlm.nih.gov/gene/568315
*tbx20*	Involved in developing the circulatory system and regulating cardiac muscle cell proliferation.	National Library of Medicine (https://www.ncbi.nlm.nih.gov/gene/57936

## Macrophages in cardiac repair, regeneration, and fibrosis

Macrophages have garnered recognition for their significant roles in the healing and scarring processes following cardiac injury, observed in both zebrafish Macrophages have garnered recognition for their significant roles in the healing and scarring processes following cardiac injury, observed in zebrafish, adult and neonatal mice and porcine models (3, 19, 25). Both, regenerative and non-regenerative animal models have the same 3 phases of cardiac repair: initial inflammatory phase followed by an anti-inflammatory phase, when cardiac proliferation occurs, and a final maturation phase. The last one is where regenerative and non-regenerative animals differ, as the first has its collagen scar reabsorbed and the other one keeps it. Inflammatory cell recruitment also occurs similarly between both types of repairs (regeneration and fibrosis). However, studies have shown that in adult mice (non-regenerative model) there is more neutrophil recruitment when comparing with neonatal mice, which is caused by decreased and delayed macrophage recruitment, resulting in an increase of the fibrotic scar (26). MI in neonatal mice and porcine hearts is mimic by permanent coronary artery ligation. These animals at that stage (1 day after birth for mice and 2 for porcine), different from adults, can regenerate their heart in a similar way that the zebrafish can, a collagen scar is formed and reabsorbed (25, 27).

Acute inflammation induced by intramyocardial injection of zymosan A, a Toll-like receptor 2 (TLR2) agonist, was shown to prevent tissue injury and increase endogenous cardiomyocyte proliferation in an adult MI injury model, which suggests that the acute immune response is sufficient to stimulate cardiomyocyte proliferation (28). TLRs are pattern recognition receptors expressed in all innate immune cells and in epithelial cells. Its classic function is to induce innate immune response by recognizing pathogen-associated molecular patterns (PAMPs), recently other functions are being elucidated, like the one mentioned above (29).

Macrophages are present throughout the repair process in different phenotypes. They initially have inflammatory features at the injury site, characteristically expressing TNFa after 24 h of injury (5, 19), and soon translate to less inflammatory profiles (5). Removal of these immune cells through different pharmacological or genetic approaches has established that macrophages are necessary for cardiomyocyte proliferation and neovascularization, and their absence led to a more significant loss of heart regenerative capacity (5, 29, 30). A study comparing two teleost fish species that present disparate outcomes after cardiac injury has demonstrated, by RNA transcriptome data analysis, that non-regenerating medaka fish (*Oryzias latipes*) have delayed and reduced macrophage recruitment, along with delayed neutrophil clearance, while regenerative zebrafish present a more robust activation of macrophages, complement system proteins, B and T cells, and increased phagocytosis (31). It has been suggested that these differences in the immune response upon cardiac cryoinjury are associated with the impaired regeneration process that occurs in medaka, in comparison with the zebrafish. Furthermore, the regenerative capacity displayed by neonatal rodents, which, upon cardiac injury, present a more significant infiltration of granulocytes and upregulation of inflammatory cytokine expression than adult rodents (32) further emphasizes the relationship between the immune response and cardiac regeneration.

Additionally, Li et al. have demonstrated that neonatal cardiac macrophages can promote cardiac repair in macrophage-depleted adult mice models after MI (30). The dichotomy between neonatal and adult macrophages was further explored in an experiment where macrophage populations were discriminated between neonatal- or adult-derived cells in a myocardial injury model. A predominance of monocyte-derived macrophages, cells considered of adult origin, was observed, attributed to a compromise in regenerative capacity and increased scar formation. In these experiments, macrophages derived from the adult spleen were transplanted into neonate mice, compromising the regenerative process (33, 34).

Macrophages in zebrafish have similar functions to those they perform in the context of cardiac injury in mice. Cardiac repair and fibrosis in mammals are well described, as mentioned above, and can be accessed in detail at Frangogiannis, 2021. Simoes et al. demonstrated that these cells actively act in collagen deposition in the zebrafish cardiac cryoinjury model at 5 days post-injury, and in mice heart infarction model (34, 35). The collagen deposition phenomenon attributed to macrophages was also mentioned in another study (6). Nonetheless, in this case, depletion of macrophages at 3 days post-injury led to more significant collagen scarring, suggesting that these cells also have a pro-resolutive function (6).

In a cardiac laser injury model in larval zebrafish, it was demonstrated that macrophages are necessary for lesion debridement since the absence of macrophages leads to the accumulation of apoptotic cardiomyocytes within 24 h post-injury (19), the lack of macrophages observed in *irf8*−/− mutants delayed lesion closure and cardiac regeneration (19). It was suggested that macrophages were not entirely needed for the functional and structural recovery of the larval heart due to the greater abundance of neutrophils that are naturally present in *irf8*−/− mutants, which likely compensated for macrophage loss. Additionally, cardiomyocyte proliferation was impaired when neutrophil recruitment was reduced by using a CXCR1/2, chemokine receptors induce neutrophil chemotaxis, antagonist (19). These data indicate that macrophages have both pro-fibrotic and pro-resolution functions. More studies are needed to unravel the extensive complexity of these cells in the context of repair and regeneration.

## RNA sequencing as a technique to study cardiac repair

### Bulk and single-cell RNA sequencing

**Figure 2:**
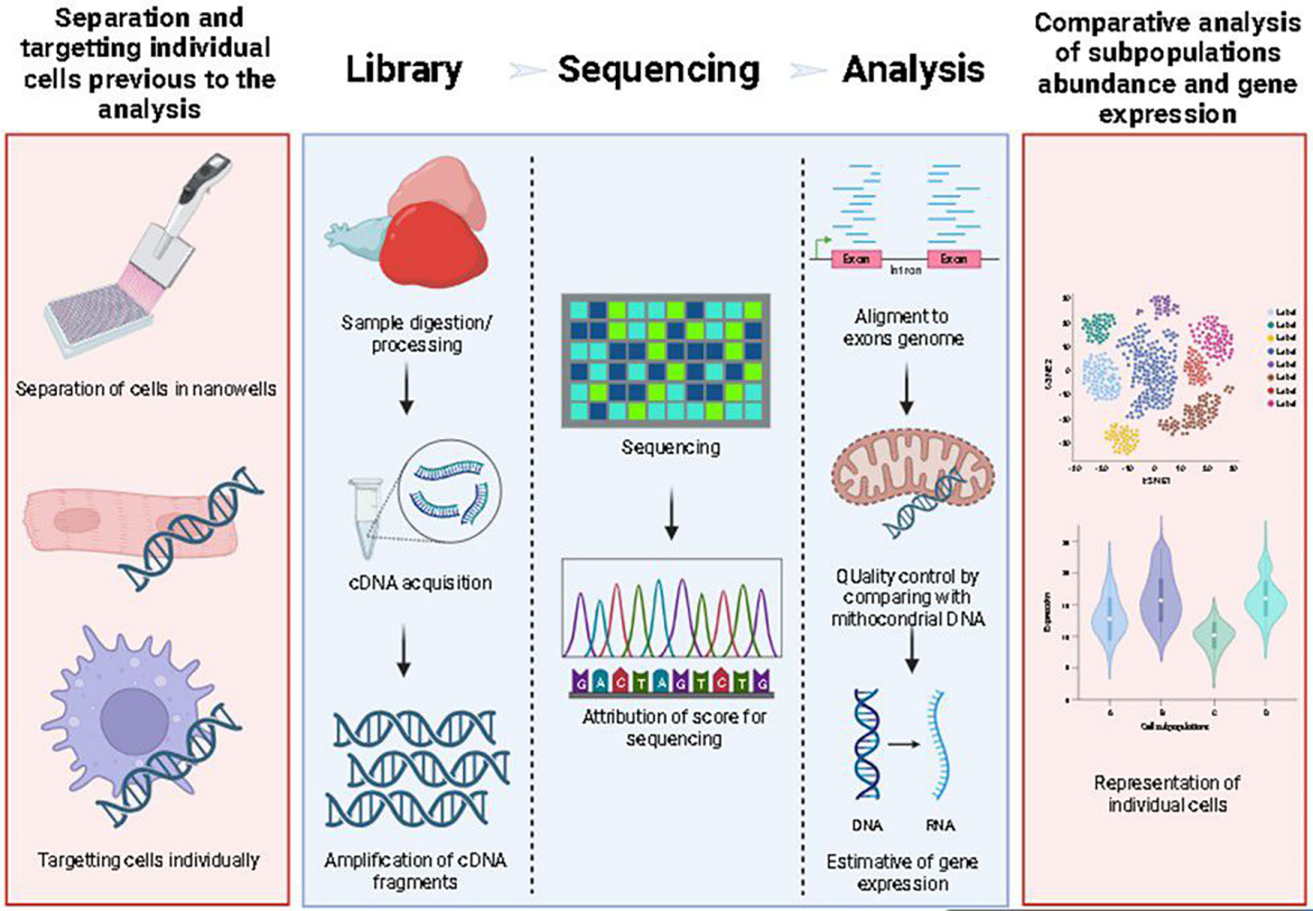
Schema of scRNAseq workflow. Red boxes are exclusive to scRNAseq, while blue boxes represent the workflow for any RNAseq technique. For scRNAseq, single cell separation and sample digestion/preparation is performed in nano wells (first box), this is done after sample processing (second box), and then, after cDNA extraction and PCR indexing, the cDNA is sequenced, aligned and analyzed.

Bulk RNA sequencing (RNAseq) is a molecular technique available for measuring the diversity and abundance of RNA fragments. To explore the global changes in gene expression in a tissue (36). In brief, an RNAseq workflow consists of 3 steps: library preparation, sequencing, and analysis. scRNAseq involves the same technique but isolates individual cells as a target. The library preparation requires individually targeting cells with specific oligonucleotides to identify gene expression in individual cells of a tissue (Figure 2). In bioinformatic analysis, identical sequences offered by the sequencing company identify each cell type. The filtration of a particular gene identifies a group of cells with similar sequences. For example, for macrophages, the tracked gene can be macrophage-expressed gene (mpeg) or microfibril-associated protein 4 (mfap4) (9).

When first described the main advantage of scRNAseq was allowing for the use of scarce/low-abundant samples; specifically, scRNAseq allowed for the determination of gene expression in a four-cell stage embryo (36). This technique is also capable of distinguishing different cell populations in multiple organs, as highlighted by the identification of dendritic cell populations in various tissues in zebrafish (9).

However, the efficiency in gathering scRNAseq data depends intensely on the technique used; different methods of scRNAseq can lead to hugely different clusterization patterns (37), which can seriously compromise comparisons among similar study designs and analysis of complex samples of multiple cell types. Also, non-specific reads can generate virtual separation of clusters, which is a defect of the technique. Differentiating cell populations only by scRNAseq could be challenging and should be preceded by a sorting step in complex samples (38). Moreover, sequencing could be shallow and indicate uncertain results (39).

## scRNAseq use to study macrophages during heart injury in zebrafish

The scRNAseq technique allows the tracking of chosen markers in different cell populations. For example, this technique was used to investigate differential expression of the gene *leptin b* among cardiac tissue cell populations (40). It can also be used to unravel intercellular communication, such as the expression of the chemokine *cxcl12a* by an epicardial subpopulation (Epi3), a crucial process related to leukocyte attraction to the heart upon injury (41). Additionally, it can be used as an exploratory tool, to describe different cell populations gene expression.

Generating these data could explain why zebrafish are more strikingly efficient in organ regeneration than other animals. Zebrafish usage presents unique advantages to scRNAseq, such as the rarity of pseudogenes, making it easier to align the human genome. Additionally, the zebrafish contains transposable element type I in 11% of its genome, compared to 44% in humans (42). On the other hand, zebrafish present transposable element type II in 39% of their genome vs. 3.2% in humans, making this a scarce element in mammals (42), which could influence the analysis of some genes to the detriment to others. The lack of pseudogenes, combined with the well-established zebrafish experimental models, such as the cryoinjury and the ventricular resection, makes scRNAseq an incredible tool for revealing specific mechanisms of cell populations.

scRNAseq data have demonstrated that macrophages represent 2.4% of the entire heart cell population in the zebrafish and 9.6% of the total non-cardiomyocyte cells. Macrophages are the fourth most abundant population (using the mpeg gene as a marker), the first being cardiomyocytes (39% of the total population), the second being endothelial cells (EC) (37% of the total heart population, but 36% of the ventricular cell population) (43), and the third erythrocytes (44). This is a similar proportion to what has been described in adult mouse during homeostasis (7, 43). However, the authors used flow cytometry technique to identify cell populations, and different markers for each cell, the classical ones used for mouse: CD31 and CD102 for EC, and CD45 and CD11b for leucocytes (45). Total immune cell numbers increase dramatically at 3 days post-cardiac apex resection and gradually decrease in quantity (6, 34, 46, 47). Still, at 30 days post cryoinjury (dp cryo), this amount is more significant than that existing in the uninjured heart, according to scRNAseq data (46). This highlights the importance of macrophages at all stages in the response to cardiac insult in zebrafish.

However, how macrophages convert through different phenotypes during the repair process remains unclear. There is a well-established marker for inflammatory macrophages, TNFa (48). TNFa-positive macrophages have been extensively studied in different lesion models. They are characteristically present at the initial repair phases (6), performing important roles during regeneration related to cell recruitment, immune system activation, debris elimination, and cell reprogramming (49), among others (8, 50). Still, this marker does not represent the diversity of these cells, because its expression is not exclusive of inflammatory macrophages, according to published data (7, 44), as discussed below. Also, there has yet to be a well-established marker for reparative macrophages. Based on scRNAseq data, a subpopulation of macrophages expressing high levels of galectin 3 binding protein b (*lgals3bpb*), a phagocytic receptor, was reported to be a pro-inflammatory marker (9). However, new studies must verify its expression in different injury scenarios, including heart injury.

Macrophages can also be divided into resident and recruited. A recent study highlighted the difference between these populations, comparing regenerative with non-regenerative hearts, through scRNAseq. By doing this, Lai and colleagues (31) discovered that two subpopulations of resident macrophages (named Mac 2 and Mac 3) are highly present in regenerative conditions and decreases in non-regenerative conditions. Mac 2 regulate NO homeostasis, control inflammation, control neutrophil migration and reverse migration, while Mac 3 present protective and regenerative functions. Additionally, the findings showed that Mac3 macrophage subpopulation transits between all other subpopulations at the cardiac regeneration. Therefore, it functions as the major controller of this process. This crucial role was later confirmed by depletion of Mac2 and Mac3 subpopulations, which led to impairment of CM recruitment, vascularization, inflammation control and fibrotic scar resolution.

Moreover, many studies have recently tried discovering a marker for reparative macrophages using transcriptomic approaches. Sanz-Morejón and colleagues suggested *wt1b* as a marker gene for reparative macrophages by comparing bulk transcriptome data from ventricular macrophages derived from fish with and without *wt1b* at 4 dp cryo. The *wt1b*-positive cells showed a greater expression of genes related to scar resolution, matrix remodeling, homeostasis, angiogenesis, leukocyte migration, and regulation of the immune response in comparison with *wt1b*-negative macrophages (51). However, *tnfa* was not differentially expressed. This data suggests that *wt1b* might be a marker gene for an intermediary macrophage with inflammatory and pro-reparative characteristics. Notably, *wt1b*-positive macrophages mostly come from the renal medulla, the zebrafish hematopoietic organ. However, despite cardiomyocyte proliferation, no change in fibrosis and deposition of fibrotic tissue was observed in the absence of *wt1b (*51).

scRNAseq analysis makes it possible to differentiate macrophage subpopulations during heart injury repair. Hong Ma and colleagues analyzed the injured zebrafish heart after 2, 7, and 14 days post-ventricular resection (dp vr) (7). They demonstrated that macrophages are the cells that change over time, and they differentiated these changes into five subpopulations. These cells alter their function dynamics over time during injury repair (7). They suggested that this is related to post-transcriptional and epigenetic control, a relevant research subject that needs to be better explored in the context of cardiac insult. Also, they described that macrophages are the most abundant non-cardiomyocyte cell population at 2 dpvr (7).

Within 2 dp vr, the initial macrophage subpopulation is an inflammatory cell that expresses classic inflammatory genes, such as *tnfa*, *il1b*, and *csf3b*, a population probably responsible for fibroblast recruitment (7). Another subpopulation described and increased at initial time points is the phagocytic subpopulation, which expresses genes associated with phagocytosis, such as *cd63*, and genes related to cathepsins. The inflammatory subpopulation remains elevated at 7 dp vr, while the phagocytic subpopulation decreases immediately (7). A third subpopulation described is related to antigen presentation and gradually increases as the lesion is repaired and becomes predominant after 14 dp vr. Furthermore, two other small subpopulations are still present, one related to debris elimination at 7 dp vr; the other is connected to proliferation and is present at 2 dp vr but decreases over time (7). This difference between research articles shows how diverse macrophages can be and the multiple roles they can perform during zebrafish cardiac regeneration. However, the question of which zebrafish macrophages feature allows them to regenerate at the expense of other species, such as humans, remains unanswered.

Another study showed different results after performing scRNAseq of macrophages following cardiac resection. Rolland and colleagues found that at 7 and 14 dp vr, there is a significant presence of activated macrophages expressing *tnfa, cd40, il1b,* and *il6r*, classical inflammatory markers (44), classified as the resident macrophages. This different gene expression can be related to various data treatments and clusterization but doesn't mean that these cells are not present at 2 dp vr, as shown by Ma and colleagues (7).

Comparing organisms with regenerative abilities with non-regenerative ones may address this central question. A recent study using scRNAseq data from uninjured 3- and 14- dp cryo ventricles from zebrafish and medaka found that interferon-induced gene, *isg15*, is present mainly in endothelial cells in the zebrafish, but not in medaka. Zebrafish and medaka present the same number of macrophages in uninjured hearts, but upon cryoinjury, zebrafish increase their number of macrophages and the surrounding endothelial *isg15* positive cells. In contrast, medaka did not increase the number of macrophages (52). Additionally, medaka macrophages express more *tnfa*, while zebrafish macrophages express more *cd9b* (related to M2-like macrophages). Thus, macrophages are necessary for heart regeneration, although a constant inflammatory state is detrimental to regeneration (6, 52). A persistent inflammatory state promotes neutrophil retention by blocking their phagocytosis by macrophages (53).

Although macrophages can promote inflammation, they can also repress this process. Therefore, bulk RNA sequencing of ventricles at 21 dp cryo following macrophage depletion with clodronate encapsulated liposomes demonstrated a permanent inflammatory state characterized by increased expression of inflammatory genes involved in the immune response, apoptosis, immune signaling, phagocytosis, leukocyte recruitment, and migration (10), and these results strengthen the importance of macrophages in downregulating the immune response.

## Macrophage profile in cardiac resection and cryoinjury

**Figure 3:**
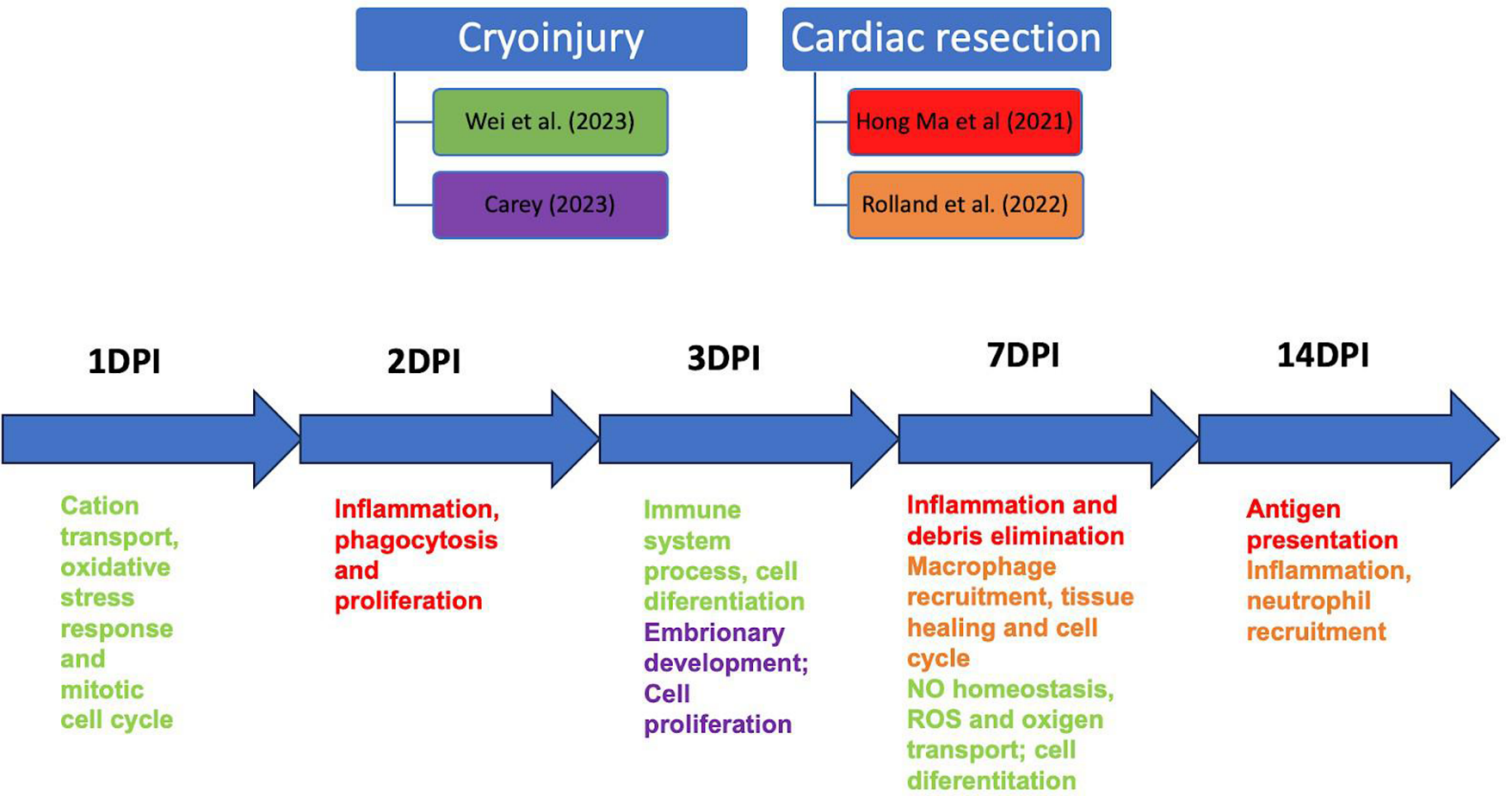
Schematic showing the relationship between studies that performed scRNAseq of macrophages in cardiac cryoinjury and cardiac resection at other time points. DPI, days post-injury.

The studies that performed scRNAseq of macrophages following zebrafish cardiac resection (7, 44) showed that these cells remain activated, performing immune system functions, such as antigen presentation, debris elimination, and cell recruitment, until 14 dp vr (Figure 3). TNFα is well well-known inflammatory gene, and it has been widely used as a marker of inflammatory macrophages. However, Carey and colleagues (52) compared macrophage expression of zebrafish with medaka fish, and they showed that macrophages equally express TNFα at 3 (an inflammatory phase of the regenerative process) and 14 dpi (a reparative phase) in zebrafish. Additionally, Rolland and colleagues showed that tnfa is a marker for the resident macrophage cluster (44). Although this does not erase the fact that this cell population can present inflammatory characteristics, together with Carey results, it shows that the current macrophage division of inflammatory macrophages as being tnfα+ and anti-inflammatory macrophages being tnf*α*- is not totally accurate, as another macrophage population can be characterized by the tnfα high expression, like resident macrophages.

Both injury models generated macrophage responses related to cell proliferation debris phagocytosis, activation of the oxidative metabolism, oxidative stress, and other related phagocytic signals, at the same repair moment. However, Rolland and colleagues described the central cluster at 3 dp vr as mostly expressing regenerative macrophage genes, which persisted at 7 dp vr (Figure 3). Another similarity is that resident macrophage clusters were present at 3 and 7 dp vr (37) and 1, 3, and 7 dp cryo, suggesting a crucial role of this macrophage profile in promoting tissue repair in both injury types.

Therefore, macrophages, following both injury types, present inflammatory, proliferative, and reparative phenotypes at 1–7 days post-injury, the central time points studied in the articles reviewed. Only two studies used the cardiac resection model to analyze the late time point of 14 days post-injury (7, 44). Hence, scRNAseq of macrophages in the context of cardiac cryoinjury and resection, in late time points, 14, 21, and more days post-injury, are needed to better understand the dynamics of the collagen scar resorption since complete regeneration occurs only after 90 dp cryo and 60 dp vr (16). However, the significant decrease in macrophage numbers after 14 days of injury at both injury types might be an experimental challenge.

Taken all reviewed data together, we can conclude that, despite minor differences regarding macrophage subsets comparing scRNAseq and bulk RNA data, they present the same function in cardiac repair. However, Nadia and colleagues (54) performed meta-analysis, using the same bioinformatic parameters, of bulk RNA sequencing data of cryoinjury, ventricular resection and genetic ablation, injury models. They found that the major differences between sequenced data is due to different sequencing platform. Additionally, they compared all 3 injured models with sham animals, and the most differentially expressed were between genetic ablation and sham animals. There were more different genes expressed between sham and uninjured animals than between cryoinjury and sham (54). As they did not focus their analysis on macrophages and in scRNAseq data, and as the differences among injury models are evident, it is crucial to perform meta-analysis of macrophages generated scRNAseq data to provide more reliable macrophage markers of cardiac regeneration and fibrosis.

Thus, disparities were identified in the analysis tools used by different authors, raising a pertinent concern: the comparative analysis between these studies may become biased, given that the compared data were processed using different tools. This concern covers the variation in the versions of the statistical packages and the divergences in the reference genomes used to produce expression matrices.

It is crucial to detail the methods and tools used in any bioinformatics analysis, as some authors need to include this essential information. Normalizing the raw data and reprocessing all scRNASeq reads using the same tools is imperative for a more reliable comparative analysis. This approach aims to obtain parameterized results under uniform conditions, contributing to the robustness and consistency of the conclusions.

Notably, the information and procedures highlighted aim to establish a more precise correlation between the results obtained by the authors referenced in this comparative review. However, it is crucial to consider that the comparisons made may only partially reflect the reality described in the reference materials since we compare results generated by different tools. Although these results may use other metrics in this context, they may express the same information.

From scRNAseq of macrophages data, new therapies can be developed. For example, chimeric antigen receptors macrophages (CAR-M) is a technology that changes macrophages receptors and can modulate this cell's activation according to what changes have been made on it (55). Therefore, some studies have been using CAR-M to target fibroblast activating protein (FAP) in the context of cardiac fibrosis. CAR-M can diminish the cardiac fibrosis, through the phagocytosis increase of fibroblasts and myofibroblasts (55). Additionally, pharmacological strategies can also be used to target and stimulate the reparative capacity of anti-inflammatory macrophages and inhibit pro fibrotic macrophages. Therefore, as more clarified the molecular bases involved in cardiac regeneration, repair and fibrosis, more efficient and specific those therapies can be (56).

## Conclusion

The zebrafish has proven to be an excellent model for studying cardiac injury, repair, and regeneration, enabling the application of various methodologies to mimic infarction scenarios. In contrast to adult mammalian hearts, zebrafish hearts regenerate rapidly following injury through cardiomyocyte dedifferentiation and proliferation (47). Notably, there is significant involvement of the acute immune response in the cardiac regeneration process (31), particularly the role of macrophages, which have shown essential participation in the removal of apoptotic cells, epicardial activation, and cardiomyocyte proliferation in zebrafish larvae (19). Similarly, the delicate balance of the inflammatory response, particularly maintaining the phenotypic state of macrophages, plays a crucial role in scar resolution and, consequently, in complete adult zebrafish heart regeneration (6). Therefore, due to their highly heterogeneous behavior, single cell sequencing techniques enable the identification and characterization of how macrophages molecularly respond to cardiac injury scenarios, allowing for a better understanding of their specific functions in physiological and pathological processes, despite the concern of a better standardization of data present in the literature. Hence, studying cardiac regeneration in animal models, such as zebrafish, can aid in identifying involved mechanisms and provide insights for developing new therapeutic targets for human cardiac injuries.
